# A Case Report of Dilacerated Crown of a Permanent Mandibular Central Incisor

**Published:** 2016-11

**Authors:** Behnam Bolhari, Salma Pirmoazen, Ensieh Taftian, Somayeh Dehghan

**Affiliations:** 1 Associate Professor, Dental Research Center, Dentistry Research Institute, Tehran University of Medical Sciences, Tehran, Iran; Department of Endodontics, School of Dentistry, Tehran University of Medical Sciences, Tehran, Iran; 2 Assistant Professor, Department of Endodontics, School of Dentistry, Tehran University of Medical Sciences, Tehran, Iran; 3 Student, Department of Surgery, School of Medicine, Tehran University of Medical Sciences, Tehran, Iran; 4 Assistant Professor, Department of Endodontics, School of Dentistry, Alborz University of Medical Sciences, Karaj, Iran

**Keywords:** Tooth, Deciduous, Tooth Avulsion, Tooth, Tooth Abnormalities

## Abstract

Trauma to primary teeth can lead to devastating sequels in development of permanent successors. The disturbance may range from enamel hypoplasia and/or hypo-calcification to arrest of dental bud development. Crown dilaceration of permanent teeth is one of the consequences of trauma to deciduous teeth mainly due to intrusion or avulsion. This report presents a mandibular central incisor with dilacerated crown and yellowish discoloration with symptomatic apical abscess. History revealed avulsion of primary mandibular central incisors. The purpose of this report is to present: 1. Reasons of dilacerated crown, yellowish discoloration and necrotic pulp in this case, 2. Treatment options in different types of crown dilacerations and also in this case. The tooth was successfully managed by nonsurgical root canal therapy and restoration with composite resin to restore esthetics. We emphasize that trauma to deciduous teeth should not be understated, and regular follow up is essential.

## INTRODUCTION

Trauma to primary teeth could leave serious consequences on permanent teeth. Some of these consequences include sequestration of permanent tooth germ, partial or complete arrest of root formation, root dilacerations, crown dilacerations, developmental disturbances of enamel (hypo-calcification and/or hypoplasia) and eruption disturbances [1]. Previous studies showed that avulsion and intrusion of primary tooth could cause more serious sequel in successor tooth [2,3]. One of these sequels is dilaceration that is defined as an abrupt deviation of the long axis of the root or crown [3]. According to previous studies, 3–9% of all sequels after traumatic injuries are crown dilacerations [3,4]. Although the etiology of dilaceration is not completely clear, it is mainly a result of acute mechanical trauma to primary tooth. This is the most acceptable hypothesis [5]. Those without a history of trauma are known as idiopathic crown dilacerations [5].

There are some challenges in diagnosis and treatment planning of teeth with dilacerated crowns. The main challenge is particular morphology of such cases; therefore, a multidisciplinary approach is necessary [6]. Also, to achieve a favorable outcome, an astute clinician should consider different aspects including taking a proper dental history and precise clinical examination in addition to appropriate radiography [7]. With regard to limitation of conventional radiography, cone beam computed tomography is helpful in more complex cases such as severe crown dilacerations [7]. Another challenge, which appears during endodontic treatment, is the angulation between the crown and the root; hence, access cavity preparation should be done with caution [6]. Crown dilacerations are less common than root dilacerations; furthermore, crown dilacerations are less prevalent in mandibular central incisors than in maxillary central incisors [6,7]. This paper presents a rare case of crown dilaceration of a mandibular central incisor with yellowish discoloration and necrotic pulp.

## CASE REPORT

**Fig. 1:**
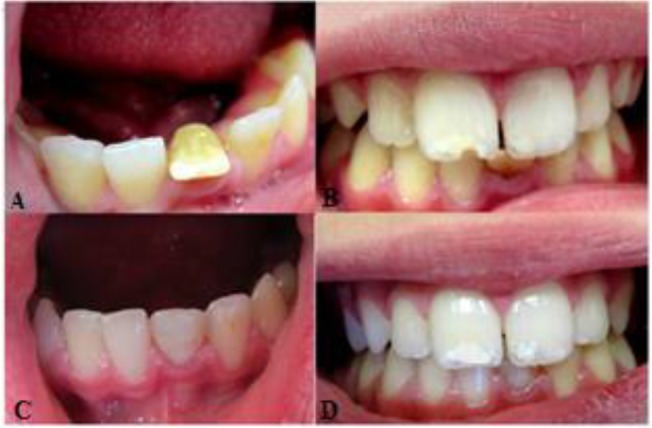
Clinical photographs demonstrating (A, B) preoperative state of mandibular left central incisor with abnormal direction due to crown dilacerations: notice the yellowish color of the tooth, (C) postoperative appearance and (D) one-year follow-up

An 11-year-old girl was referred to our Department (Department of Endodontics, Faculty of Dentistry, Tehran University of Medical Sciences, Tehran, Iran) in June 2012. Her parents complained of “an abscess related to her lower anterior teeth developed one month ago and she took antibiotics for it”. She had no systemic problem. Dental history revealed a previous trauma to primary mandibular anterior teeth (teeth #71, 81) when she was three years old. Her primary central incisors were avulsed due to that trauma. Avulsed primary teeth had not been replanted. Clinical examination revealed a mandibular left central incisor (tooth #31) with moderate dilaceration of crown. The tooth had a labial angulation and the region of dilaceration was in the cervical part of the tooth. The crown showed yellowish discoloration, which was assumed to be due to enamel hypocalcification (Fig. 1a, 1b]. The tooth had no caries. Vitality tests including cold test (Kalte Spray; Marcadent GmbH, Erkelenz, Germany), heat test (by a low-speed rubber cup) and electric pulp test (Coxo Dental Pulp Test C-Pulse; Soin Dental Co., West Byfleet, UK) were done.

**Fig. 2:**
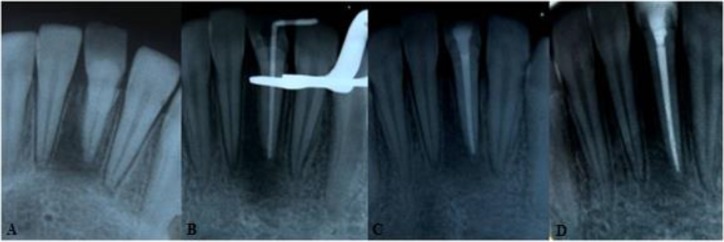
Images showing (A) preoperative X-ray, (B) X-ray during treatment, (C) postoperative X-ray and (D) one-year follow-up

The tooth did not respond to vitality tests while the adjacent teeth responded normally. Patient occlusion was class I with moderate bite. In periodontal examination, the probing depth of the tooth was normal (< 3mm) with normal mobility and no sensitivity to percussion or palpation. Radiographic evaluation revealed periapical radiolucency associated with the left central incisor; the apex was closed (Fig. 2a). Although other teeth had a normal appearance on radiographs, the tooth image was relatively shortened and was more opaque around the cingulum. Based on clinical and radiographic findings, crown dilaceration with enamel hypocalcification and necrotic pulp with asymptomatic apical periodontitis was diagnosed. According to this diagnosis, endodontic treatment was initiated. The tooth was anesthetized with left mental nerve block using 2% lidocaine with 1:80,000 epinephrine (Persocaine-E; Daroupakhsh pharmaceutical Mfg. Co., Tehran, Iran). Access cavity was prepared using a high-speed fissure diamond bur (# 010). Since the axis of the crown and the axis of the root are different in teeth with dilacerated crowns, caution was attempted during access cavity preparation. Bone contour palpation as well as exploration of cervical area of the tooth with an explorer were done. In this case, due to labial position of the crown, access cavity was moved from the middle third toward the cingulum. For preparation of the outline, the bur was positioned perpendicular to the cingulum. For exposure of the pulp, the angle of the bur was changed to parallel to the long axis of the root. Then, the tooth was isolated with rubber dam while the tooth was draining through the canal. Working length was determined using a #15 K-file (Dentsply Maillefer, Ballaigues, Switzerland) and electronic apex locator (Root ZX; J Morita Corp., Osaka, Japan) and confirmed with radiography. Root canal was prepared to the apical size #40 by Mtwo rotary instruments (VDW, Munich, Germany) according to the manufacturer’s instructions.

Canal was irrigated with 5.25% sodium hypochlorite and saline in-between filings. Calcium hydroxide powder (Kimia Dent Co., Tehran, Iran) was mixed with normal saline and placed in the canal. After one week, canal was obturated with gutta-percha (Aria Dent, Tehran, Iran) and AH26 (Dentsply, DeTrey, Germany) sealer using lateral compaction technique (Fig. 2b, 2c).

After removing the incisal third of the crown, labial and lingual contours were modified using a high-speed chamfer diamond bur. For better retention, intracanal gutta-percha was removed by 3mm apical to the cementoenamel junction. After enamel and dentin preparation, esthetic restoration with a light-cure composite resin was performed (Filtek™ Z350; 3M ESPE, St. Paul, MN, USA) (Fig. 1c).

Careful examination and occlusal adjustment were done. The patient was followed up for six and 12 months. According to the clinical examination in the follow-ups, there was no sensitivity in percussion or palpation and no mobility; the periodontium was healthy. Radiographic evaluation revealed that periapical radiolucency had healed (Figs.1d, 2d).

## DISCUSSION

In case of trauma to primary teeth, we have to spend enough time to find the sequels of trauma to permanent tooth germ; therefore, we should thoroughly follow the patient for a long period of time.

Trauma to primary teeth can lead to devastating sequels in permanent successors. The sequels vary from yellowish brown discoloration and/or developmental disturbances (hypocalcification and/or hypoplasia) to an arrest in permanent bud development [6]. Several factors can determine the adverse effects of trauma to primary teeth on permanent teeth. These factors include type of trauma (intensity and direction) to primary teeth, age of child at the time of trauma, relationship between the apices of primary teeth and buds of permanent successors and stage of root formation of permanent tooth [4,8].

Crown dilaceration of permanent teeth is generally the consequence of avulsion or intrusion of their primary ancestors [8]. It usually happens between the ages of one to three years and rarely occurs after that [1]. In the present case, severe trauma (avulsion) to the mandible at the region of primary central incisors at the age of three was the reason for crown dilaceration of tooth #31.

The first issue that needs to be addressed is the etiology of yellowish discoloration of the tooth. For this purpose, first we need to review the definition of “hypoplasia” and “hypocalcification”. Enamel hypoplasia is defined as interrupted formation of enamel while enamel hypocalcification is disturbed maturation of enamel matrix [9]. Hypocalcification happens more frequently than hypoplasia [9]. In hypocalcification, tooth color may vary from white to yellow but the enamel surface is usually not defective [9].

Hypoplasia may range from pits in the enamel to serious malformation of the crown [9]. In the present case, yellowish discoloration might be a result of distortion of the ameloblastic layer induced by trauma leading to disturbed enamel maturation [7]. In addition, Altun et al, [10] discussed that bleeding following primary tooth avulsion may cause entrapment of hemoglobin products in developing enamel and result in its yellow-brownish discoloration. This might explain the yellowish discoloration in our present case. Further studies in this regard are recommended.

There is some doubt about etiologic factors causing pulp necrosis in this tooth. Katz-Sagi et al, [11] and Bassiouny et al, [12] found unusual canal obliteration after trauma to their associated primary teeth. Moreover, von Gool [9] reported that all dilacerated teeth in his study appeared to be non-vital but he could not explain the reason for non-vitality of these teeth. On the other hand, Nasry and Barclay [13] and Yasa and Arslan [14] have shown that occlusal trauma is responsible for the pathosis and risk of pulpal and periradicular diseases especially when teeth are badly aligned [14]. According to another study, microorganisms can invade the pulp space through defective enamel leading to pulp necrosis [15].

Andreasen et al, [16] concluded that the dilacerated area of crown is free of gingiva and provides a pathway for bacterial invasion. In conclusion, pulp necrosis of the present case might be due to these reasons: 1- The protruded position of the tooth with dilacerated crown made it susceptible to subsequent trauma and resulted in pulp necrosis (more likely). 2- Developmental disturbances of the tooth (hypocalcification and crown dilaceration) provided an opportunity for microorganisms’ invasion leading to pulp necrosis. Crown dilacerations are categorized into three groups according to their eruption status namely totally impacted, partially erupted and totally erupted. Based on this, treatment options vary. For totally impacted dilacerated crowns, surgical exposure with/without orthodontic treatment and esthetic periodontal surgery (if required) have been recommended with considerable success [6]. In partially erupted cases, surgical extrusion is usually recommended [17]. In totally erupted cases [7], if the angle of deviation between the root and the crown is not severe, buccal and/or palatal contouring and reconstruction by composite resin is the preferred treatment.

The most important difficulty encountered in such cases is their complex treatment planning; therefore, an interdisciplinary approach is necessary [6]. Another challenge, which appears during endodontic treatment, is angulation of the linear relationship of the crown and root. To avoid procedural errors, precise clinical examination should be performed before access cavity preparation. For this purpose, palpation of bone contour of the root and exploration of cervical area of the tooth with an explorer can be helpful [18].

Von Gool [9] reported that teeth with dilacerated crowns in the mandible erupt in 75% of the cases; in contrast, maxillary teeth with dilacerated crowns often remain unerupted. In the present case, since the tooth was erupted labially, we managed it by endodontic and restorative approach. It should be pointed out that since the success rate in these cases depends on periodontal health, careful monitoring is extremely important.

## CONCLUSION

Trauma to primary teeth must be followed regularly because of possible future complications. To achieve desirable esthetics, function as well as healthy periodontium of teeth with crown dilaceration, correct diagnosis and appropriate interdisciplinary treatment strategy are mandatory. In moderate crown dilacerations, endodontic and conservative restorative treatment with periodic follow-ups could lead to a favorable outcome.
